# Development of a psychological management intervention protocol for Elderly Patients with Chronic Diseases based on the Empowerment Theory: A Delphi study

**DOI:** 10.1371/journal.pone.0335076

**Published:** 2025-12-30

**Authors:** Wenting Su, Hongju Yang, Chengyan Fu, Hanzong Yang

**Affiliations:** 1 School of Nursing, Shandong First Medical University & Shandong Academy of Medical Sciences, Taian, China; 2 Department of Nursing, the Second Affiliated Hospital of Shandong First Medical University, Taian, China; 3 Shanxi Normal University, Shanxi, China; Instituto Mexicano del Seguro Social, MEXICO

## Abstract

**Objective:**

This study aims to establish a psychological intervention program for elderly patients with chronic diseases based on the theory of empowerment.

**Methods:**

Through literature review, current situation surveys, and group discussions, we drafted the initial version of a psychological intervention program based on the empowerment theory. The final version of the program was developed through two rounds of Delphi expert consultations via letters.

**Results:**

The response rates for two rounds both were 100% (n = 19), with an authority coefficient of 0.87. The coefficients of variation ranged from 0.05 to 0.24, and the Kendall’s W coefficient was 0.123 (P < 0.001). Ultimately, an index system comprising 7 primary items, 25 secondary items, and 98 tertiary items was established.

**Conclusion:**

The psychological intervention program for elderly patients with chronic diseases based on empowerment theory is scientifically sound, specific and practical. It can provide valuable references for the psychological care of elderly patients with chronic diseases and the improvement of their self-management strategies.

## Introduction

According to the latest data from the National Bureau of Statistics, the proportion of people aged 60 and above in China has reached 21.1% of the total population, and the size of the elderly group continues to expand [1]. Against this background, chronic diseases have become a core issue affecting the health of the elderly—approximately 75% of the elderly suffer from at least one chronic disease, and more than half of the elderly suffer from multiple chronic diseases simultaneously [2]. The long-term nature and complexity of chronic diseases not only impair the physiological functions of the elderly but also pose continuous challenges to their psychosocial adaptation, often leading to many problems such as Depression, Anxiety, sense of disability and Isolation [3,4]. At present, psychological interventions for elderly patients with chronic diseases are mostly limited to health education or routine psychological counseling, lacking personalized intervention programs that focus on enhancing internal strength as the core [5].

Empowerment theory aims to stimulate individual subjective initiative, and its core lies in enhancing patients’ psychological coping ability and helping patients master emotion management skills [6,7]. The empowerment process usually covers five key links: problem identification, emotional expression, goal setting, plan formulation and effect evaluation, and is committed to improving patients’ Adaptive skills from the cognitive, emotional and behavioral dimensions [8]. In recent years, more and more studies have begun to focus on the important impact of internal psychological qualities on the health of the elderly. For example, studies by Prabhakar and Tiwari [9,10] found that positive psychological traits can significantly alleviate negative emotions and improve life satisfaction. This further highlights the practical value of improving the psychological resilience of elderly patients through empowerment.

Although the empowerment theory has shown certain application potential in multiple fields, there is still a lack of psychologically empowering intervention programs with clear structure and operability for elderly patients with chronic diseases. Therefore, based on the empowerment theory, this study intends to construct a targeted psychological intervention program for elderly patients with chronic diseases through the Delphi expert consultation method, so as to fill the gap in existing services and provide theoretical basis and practical reference for improving the mental health level of this group.

## Materials and Methods

### Establishment of the Research Team

The research team consisted of 7 members, including a postgraduate supervisor, an expert in psychology, two supervising nurses, and three nursing postgraduate students. All team members collaboratively drafted the initial intervention plan.

### Selecting delphi experts

By using purposive sampling, this study invited 19 experts from various disciplines such as psychology, nursing, geriatrics, and mental health to ensure the comprehensiveness of the research perspective. The selection criteria for the experts were as follows:

(1) Holding an intermediate or higher professional title;
(2) Possessing a bachelor’s degree or higher;
(3) Having more than 10 years of work experience with extensive clinical expertise;
(4) Working in clinical, nursing, or psychological roles related to Elderly Patients with Chronic Diseases.

### Delphi expert consultation

#### Literature review.

**Fig 1:**
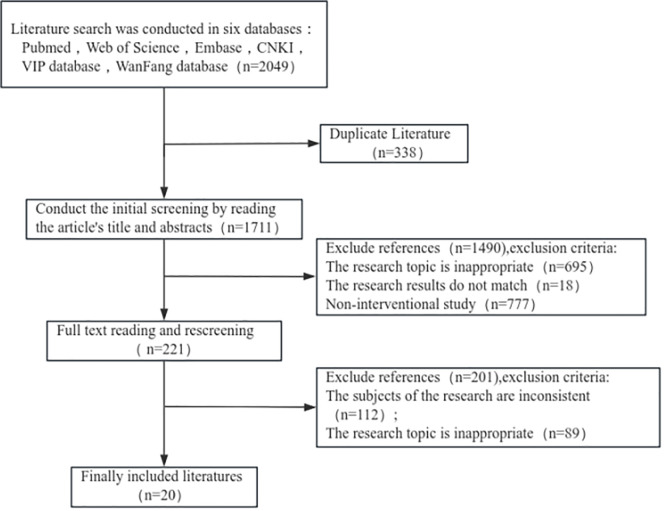
Flowchart of literature retrieval and screening.

We conducted a comprehensive examination of pertinent studies across several esteemed databases, including PubMed, Cochrane, Excerpta Medica Database (EMBASE), China National Knowledge Infrastructure (CNKI), and Wanfang Data Information Service platform. Our search methodology incorporated a combination of both keywords and medical subject heading (MESH) terms, encompassing phrases such as “elderly patients/high-age patients/psychological problems” “negative emotions/psychological distress/negative feelings/emotional disorders”“psychological intervention/psychological nursing/emotion management”. The time limit for retrievalextended from the inceptionof the databases up to January 2025. Ultimately, we identified and included 20 relevant pieces of literature (Fig 1).

### Current Status Investigation

The “Primary Screening Scale for Psychological Problems of Inpatients in General Hospitals” independently developed by the research group was adopted to investigate elderly patients with chronic diseases who met the inclusion and exclusion criteria, and analyze the psychological problems of the patients and their influencing factors. This scale has good reliability and validity, with Cronbach’s α being 0.933. The investigation results show that elderly patients with chronic diseases generally have emotions such as anxiety, depression, fear, loneliness, anger, despair and helplessness, among which anxiety, depression and fear are particularly prominent.

### Group discussion

In the group discussion, all members participated and developed the initial draft of the intervention plan together. The initial draft covered the intervention process, objectives, and intervention measures, etc. The group members reached a consensus through discussion. Based on the discussion results, the researcher sorted out and optimized the initial draft, and finally finalized the initial draft of the intervention plan.

**Formation of a Delphi expert consultation questionnaire.**

Following the principles of the Delphi method and the needs of this study, the consultation questionnaire was structured into three parts:

a. A basic information questionnaire for experts, including name, gender, length of service, and title.
b. An expert suggestion form regarding the intervention measures, which included the content of the first draft of the intervention program. The importance of each entry was scored using a 5-point Likert scale, with spaces provided for modifications, deletions, or additions.
c. Expert self-evaluation form: including two parts, the expert’s familiarity with the Delphi content and the basis for judgment.

### Questionnaire distribution

The Delphi questionnaires were distributed via email, ensuring an interval of at least two weeks between each round of consultation. After completion of the first round, the research team summarized, organized, and analyzed the experts’ opinions, based on which the second-round questionnaire was developed. The selection criteria for the items were defined as mean importance score >3.50, a coefficient of variation less than 0.25, and a full score rate higher than 20%. [11]. After two rounds of consultation, the experts’ opinions reached consensus, and the Delphi process was terminated.

### Ethical consideration

This study focuses on scheme construction, mainly covering theoretical analysis, model design and method development. During the research process, no human subjects, animal experiments or collection and use of sensitive personal information were involved. All participants were informed of the purpose of the Delphi study and gave their written consent. To protect the privacy of the participants, each participant was given a unique non-identifying code, and identifying data were not used in the result presentation.

### Statistical methods

After the questionnaire collection was completed, a database was established using SPSS 26.0 software, and the double-entry method was adopted. The measurement data were expressed as mean ± standard deviation, and the count data were expressed as frequency and percentage. The response rate of experts was represented by two rounds of questionnaires, the authority level of experts was indicated by the authority coefficient (Cr), and the formula was Cr = (Ca + Cs)/ 2. A Cr value exceeding 0.7 indicated an acceptable level of authority. The concentration of experts was represented by the mean of importance allocation (M), the coordination of experts was represented by the coefficient of variation (CV) and Kendall coefficient (ω), and CV ≤ 0.25 indicated that the experts’ opinions were highly consistent.

## Results

### General Information of Experts

In this study, a total of 19 experts completed two rounds of surveys. The experts mainly worked in fields such as psychology, geriatrics, and nursing, with an average age of 49.05 ± 5.93 years. Seven experts held a master’s degree or above (36.8%), and 17 experts held a senior professional title or above. as shown in Table 1.

**Table 1:** Basic information of experts.

Variables	N (%)
**Age**	
30 ~ 40	3(15.8)
41 ~ 50	7(36.8)
≥ 51	9(47.4)
**Level of education**	
Undergraduate	12(63.2)
Master	5(26.3)
Doctor	2(10.5)
**Title**	
Intermediate	2(10.5)
Deputy senior	10(52.6)
Senior	7(36.8)
**Work years**	
10 ~ 20	3(15.8)
21 ~ 30	7(36.8)
More than 30 years	9(47.4)
**Specialty**	
Psychology education	6(31.6)
Geriatric nursing	3(15.8)
Nursing management	4(21.0)
Nursing education	6(31.6)

### Expert Activity Level

Both rounds of consultation questionnaires were fully recovered, with an effective recovery rate of 100%. In the first round, 10 experts put forward revision suggestions (with a suggestion rate of 52.6%), and in the second round, 6 experts provided feedback (with a suggestion rate of 31.6%), indicating that the experts maintained a high level of attention and active participation in this study.

### Expert authority coefficient

The judgment coefficient (Ca) and the index familiarity (Cs) were 0.90 and 0.83, respectively, resulting in an authority coefficient (Cr) of 0.87. An authority coefficient above 0.7 is generally considered to indicate a high level of authority [12]. Therefore, the experts in this study demonstrated a high degree of authority and credibility.

### Expert concentration

In the two rounds of expert enquiries, the average scores of the importance of each project were between 3.63 and 5.00, and between 4.11 and 4.95 respectively. It is generally considered that an average score higher than 3.50 is usually acceptable, which indicates that after the adjustment of experts’ opinions and suggestions during the enquiry process, a higher consensus has been reached.

### Expert coordination coefficient

Kendall’s coefficient of concordance (ω) is used to test the consistency of experts’ scoring results for a certain item, with a value range of 0–1. The closer the ω value is to 1, the higher the degree of coordination of experts’ opinions. A lower coefficient of variation value indicates a higher degree of coordination of experts’ opinions. In this study, the CV values of the two rounds of expert consultation were 0.00–0.37 and 0.05–0.24, respectively; the Kendall’s coefficients of concordance were 0.115 and 0.123 (P < 0.001), respectively. Moreover, the ω value of the second round of consultation was higher than that of the first round, indicating that with the increase in the number of consultations, the degree of coordination of experts’ opinions on all items was good. As shown in Table 2.

**Table 2:** Coordination coefficient (ω) of expert opinions and test results.

Inquiry Round		*W*	*X* ^*2*^	*P*
First round	Primary item	0.338	38.574	*P* < 0.001
	Secondary item	0.079	50.736	*P* = 0.032
	Tertiary item	0.083	167.988	*P* < 0.001
	Overall items	0.115	324.205	*P* < 0.001
	Primary item	0.140	15.968	*P* = 0.014
Second round	Secondary item	0.084	54.153	*P* = 0.015
	Tertiary item	0.128	235.774	*P* < 0.001
	Overall items	0.123	325.298	*P* < 0.001

### Index revision

During the two rounds of inquiries, some experts put forward suggestions and opinions on the issues. The main changes are as follows:

a. Revised indicators: In each dimension, the original “face-to-face interview” was uniformly revised to “conduct face-to-face interviews with patients and their dependents” to more comprehensively assess the family support system; and the intervention strategy was uniformly adjusted to “develop intervention methods according to the severity of patients’ psychological problems” to enhance individualization and clinical applicability. These modifications conform to the scientific principles of psychology and clinical practice, can better meet the personalized needs of patients, and improve the scientificity and effectiveness of intervention. Therefore, expert opinions were adopted.
b. Added indicators: Specific behavioral descriptions are added in the behavioral observation part to enhance operability; a new goal of “patients actively seeking social support” is added in the “loneliness” dimension, reflecting social connection as an important way of psychological empowerment; at the same time, some evaluation indicators are refined to more comprehensively reflect the dynamic changes of mental health.

Following the second round of expert consultation, hypnotherapy was added to the intervention plans for the anxiety and depression dimensions based on expert feedback. After both rounds of expert consultation, a final intervention plan was established, which includes 7 primary indicators, 35 secondary indicators, and 98 tertiary indicators. See Table 3.

**Table 3:** Expert consultation results on psychological management for Elderly Chronic Disease Inpatients.

Items	Importance (score)	CV	Full score ratio	Items	Importance (score)	CV	Full score ratio
**1 Anxiety**	4.89 ± 0.32	0.06	0.89	**4 Anger**	4.47 ± 0.84	0.19	0.63
1.1 Establishment of anxiety issues	4.84 ± 0.38	0.08	0.84	4.1 Establishment of anger issues	4.79 ± 0.54	0.11	0.84
1.1.1 Interviews with patients and their families	4.89 ± 0.32	0.06	0.89	4.1.1 Conduct interviews with patients and their families	4.79 ± 0.54	0.11	0.84
1.1.2 Behavioral observation (dysphoria, difficulty concentrating)	4.84 ± 0.38	0.08	0.84	4.1.2 Behavioral observation (conflicts, pounding, etc.)	4.74 ± 0.56	0.12	0.79
1.1.3 Scale* screening and severity determination	4.58 ± 0.69	0.15	0.68	4.1.3 Screening with scales* and determination of anger level	4.47 ± 0.91	0.20	0.68
1.2 Establishing a counseling – client relationship	4.79 ± 0.42	0.09	0.09	4.2 Establish a counseling – client relationship	4.68 ± 0.67	0.14	0.79
1.2.1 Respect for patients	4.89 ± 0.32	0.06	0.89	4.2.1 Respect the patients	4.84 ± 0.38	0.08	0.84
1.2.2 Empathy	4.89 ± 0.32	0.06	0.89	4.2.2 Show empathy	4.84 ± 0.38	0.08	0.84
1.2.3 Introduction of psychological support resources	4.58 ± 0.84	0.18	0.74	4.2.3 Introduce available psychological support resources	4.53 ± 0.96	0.21	0.74
1.2.4 Introduce the manifestations and impacts of anxiety	4.37 ± 1.01	0.23	0.58	4.2.4 Introduce the manifestations and impacts of anger	4.42 ± 1.02	0.23	0.63
1.3 Set goals for anxiety intervention	4.79 ± 0.42	0.09	0.09	4.3 Set anger intervention goals	4.53 ± 0.70	0.15	0.63
1.3.1anxiety score＜5 points.	4.68 ± 0.67	0.14	0.79	4.3.1 Anger score < 5 points	4.53 ± 0.70	0.15	0.63
1.3.2 Enable patients to master 2–3 skills to cope with anxiety.	4.68 ± 0.48	0.10	0.68	4.3.2 Effectively express anger without excessive behaviors	4.63 ± 0.60	0.13	0.68
1.4 Anxiety intervention plan	4.95 ± 0.23	0.05	0.05	4.4 Anger intervention plan	4.74 ± 0.56	0.12	0.79
1.4.1 Use narrative nursing for patients with grade Ⅰ anxiety.	4.63 ± 0.60	0.13	0.68	4.4.1 For grade Ⅰ (mild) anger, use an intervention method combining narrative nursing and deep – breathing training	4.63 ± 0.68	0.15	0.74
1.4.2 Adopt an intervention approach combining narrative nursing, mindfulness therapy, and peer support for patients with grade Ⅱ anxiety.	4.68 ± 0.48	0.10	0.68	4.4.2 For grade Ⅱ (moderate) anger, use an intervention method combining narrative nursing, mindfulness breathing training, and writing emotional diaries	4.74 ± 0.56	0.12	0.79
1.4.3 Implement comprehensive intervention including narrative nursing, mindfulness therapy, peer support, and hypnotherapy for patients with grade Ⅲ anxiety. If the intervention effect is poor, invite a consultation from the psychology department and, if necessary, supplement with drug therapy.	4.63 ± 0.76	0.16	0.74	4.4.3 For grade Ⅲ (severe) anger, use a comprehensive intervention including narrative nursing, mindfulness breathing training, and NLP therapy (such as “emotional coffee machine”)	4.74 ± 0.56	0.12	0.79
1.5 Effect evaluation	4.84 ± 0.38	0.08	0.08	4.5 Effect evaluation	4.79 ± 0.54	0.11	0.84
1.5.1 The anxiety symptoms disappear or the anxiety score is < 5 points.	4.74 ± 0.56	0.12	0.79	4.5.1 Stable emotions with a score < 5 points	4.68 ± 0.58	0.12	0.74
1.5.2 Actively cooperate with the treatment and improve the quality of diet and sleep.	4.74 ± 0.45	0.10	0.74	4.5.2 No aggressive behaviors occur	4.74 ± 0.56	0.12	0.79
**2 Depression**	4.84 ± 0.38	0.08	0.84	**5 Despair**	4.63 ± 0.76	0.16	0.74
2.1 Establishment of depression problems	4.84 ± 0.38	0.08	0.08	5.1 Establishment of the despair problem	4.68 ± 0.67	0.14	0.79
2.1.1 Interviews with patients and their families	4.89 ± 0.32	0.06	0.89	5.1.1 Conduct interviews with patients and their families	4.74 ± 0.56	0.12	0.79
2.1.2 Behavioral observation (loss of interest, sleeplessness, etc.)	4.89 ± 0.32	0.06	0.89	5.1.2 Behavioral observation (low mood, self – denial)	4.79 ± 0.54	0.11	0.84
2.1.3 Use scales* for initial screening of depression and determination of its severity	4.79 ± 0.54	0.11	0.84	5.1.3 Preliminary screening with a scale* and determination of the degree of despair	4.47 ± 0.91	0.20	0.68
2.2 Establish a therapeutic relationship	4.84 ± 0.38	0.08	0.08	5.2 Establish a counseling – client relationship	4.63 ± 0.76	0.16	0.79
2.2.1 Respect the patients	4.84 ± 0.38	0.08	0.84	5.2.1 Respect the patients	4.11 ± 0.99	0.24	0.53
2.2.2 Show empathy	4.84 ± 0.38	0.08	0.84	5.2.2 Show empathy	4.89 ± 0.32	0.06	0.89
2.2.3 Introduce psychological support resources	4.63 ± 0.83	0.18	0.79	5.2.3 Introduce psychological support resources	4.68 ± 0.75	0.16	0.79
2.2.4 Introduce the manifestations and impacts of depression	4.47 ± 1.02	0.23	0.68	5.2.4 Introduce the manifestations and impacts of despair	4.53 ± 0.96	0.21	0.68
2.3 Set goals for depression intervention	4.74 ± 0.45	0.10	0.74	5.3 Set goals for despair intervention	4.63 ± 0.68	0.15	0.74
2.3.1 Depression score < 7 points	4.74 ± 0.56	0.12	0.79	5.3.1 Despair score < 5 points	4.68 ± 0.58	0.12	0.74
2.3.2 No suicidal or self – harm behaviors	4.84 ± 0.50	0.10	0.89	5.3.2 The patient is full of confidence in disease treatment	4.68 ± 0.67	0.14	0.79
2.4 Depression intervention plan	4.89 ± 0.32	0.06	0.89	5.4 Despair intervention plan	4.68 ± 0.58	0.12	0.74
2.4.1 Use narrative nursing methods for patients with grade Ⅰ depression	4.79 ± 0.54	0.11	0.84	5.4.1 Use narrative nursing for grade Ⅰ despair	4.79 ± 0.54	0.11	0.84
2.4.2 For patients with grade Ⅱ depression, adopt an integrated intervention approach combining narrative nursing, exercise therapy, music therapy, and peer support	4.68 ± 0.95	0.20	0.84	5.4.2 Use narrative and peer support for grade Ⅱ despair	4.79 ± 0.54	0.11	0.84
2.4.3 For patients with grade Ⅲ depression, implement comprehensive interventions including narrative nursing, exercise therapy, music therapy, peer support, and hypnotherapy. If the intervention effect is poor, invite a consultation from the psychology department and, if necessary, supplement with drug treatment	4.89 ± 0.32	0.06	0.89	5.4.3 Use narrative nursing, peer support, writing gratitude diaries, and the NLP gold – dust method for grade Ⅲ despair	4.26 ± 0.93	0.22	0.58
2.5 Effect evaluation	4.84 ± 0.38	0.08	0.84	5.5 Effect evaluation	4.63 ± 0.68	0.15	0.74
2.5.1 Alleviate depression and restore vitality	4.79 ± 0.42	0.09	0.79	5.5.1 Score < 5 points and rebuild confidence in rehabilitation	4.84 ± 0.50	0.10	0.89
2.5.2 No suicidal tendencies or self – harm behaviors	4.74 ± 0.65	0.14	0.84	5.5.2 The patient clarifies future plans	4.53 ± 0.91	0.20	0.74
**3 Fear**	4.32 ± 0.95	0.22	0.58	**6 Loneliness**	4.26 ± 0.81	0.19	0.47
3.1 Establishment of fear issues	4.79 ± 0.54	0.11	0.84	6.1 Establishment of the loneliness problem	4.74 ± 0.45	0.10	0.74
3.1.1 Conduct face-to-face interviews with patients and their families	4.84 ± 0.38	0.08	0.84	6.1.1 Conduct interviews with patients and their families	4.84 ± 0.38	0.08	0.84
3.1.2 Behavioral observation (avoidance, crying, screaming)	4.79 ± 0.42	0.09	0.79	6.1.2 Behavioral observation (withdrawal, restlessness, etc.)	4.84 ± 0.38	0.08	0.84
3.1.3 Initial screening with a scale* and determination of the degree of fear	4.58 ± 0.77	0.17	0.74	6.1.3 Preliminary screening with a scale* and determination of the degree of loneliness	4.58 ± 0.84	0.18	0.74
3.2 Establish a counseling-client relationship	4.68 ± 0.67	0.14	0.79	6.2 Establish a counseling – client relationship	4.58 ± 0.69	0.15	0.68
3.2.1 Respect the patients	4.84 ± 0.38	0.08	0.84	6.2.1 Respect the patients	4.84 ± 0.38	0.08	0.84
3.2.2 Show empathy	4.84 ± 0.38	0.08	0.84	6.2.2 Empathy	4.26 ± 0.93	0.22	0.58
3.2.3 Introduce psychological support resources	4.53 ± 0.96	0.21	0.74	6.2.3 Introduce psychological support resources	4.26 ± 0.93	0.22	0.58
3.2.4 Introduce the manifestations and impacts of fear	4.53 ± 0.96	0.21	0.68	6.2.4 Introduce the manifestations and impacts of loneliness	4.26 ± 0.93	0.22	0.58
3.3 Set goals for fear intervention	4.74 ± 0.56	0.12	0.79	6.3 Set goals for loneliness intervention	4.63 ± 0.60	0.13	0.68
3.3.1 Fear score < 7 points	4.63 ± 0.68	0.15	0.74	6.3.1 Loneliness score < 4 points	4.26 ± 0.93	0.22	0.58
3.3.2 Calmly face the disease or treatment	4.74 ± 0.45	0.10	0.74	6.3.2 Search for social resources and obtain support	4.26 ± 0.93	0.22	0.58
3.4 Fear intervention plan	4.84 ± 0.50	0.10	0.88	6.4 Loneliness intervention plan	4.63 ± 0.60	0.13	0.68
3.4.1 Use narrative nursing for grade Ⅰ fear	4.74 ± 0.56	0.12	0.79	6.4.1 Use narrative nursing for grade Ⅰ loneliness	4.74 ± 0.56	0.12	0.79
3.4.2 Use narrative, breathing relaxation, and peer support for grade Ⅱ fear	4.79 ± 0.42	0.09	0.79	6.4.2 Use narrative and peer support for grade Ⅱ loneliness	4.79 ± 0.42	0.09	0.79
3.4.3 Use narrative nursing, NLP fear elimination method, and progressive muscle relaxation training for grade Ⅲ fear	4.79 ± 0.42	0.09	0.79	6.4.3 Use narrative nursing, peer support, and NLP therapy (self – acceptance method) for grade Ⅲ loneliness	4.79 ± 0.42	0.09	0.79
3.5 Effect evaluation	4.79 ± 0.54	0.11	0.84	6.5 Effect evaluation	4.63 ± 0.60	0.13	0.68
3.5.1 The sense of fear is reduced, and the score < 7 points	4.79 ± 0.54	0.11	0.84	6.5.1 Loneliness is alleviated, with a score < 4 points	4.21 ± 0.92	0.22	0.53
3.5.2 Bravely face the disease and treatment	4.74 ± 0.56	0.12	0.79	6.5.2 Develop interests and hobbies and obtain support	4.16 ± 0.96	0.23	0.53
**7 Helplessness**	4.42 ± 0.84	0.19	0.58	7.3 Develop intervention goals for helplessness	4.79 ± 0.42	0.09	0.79
7.1 Establishment of the helplessness problem	4.89 ± 0.32	0.06	0.89	7.3.1 Helplessness score < 5 points	4.63 ± 0.68	0.15	0.74
7.1.1 Conduct face-to-face interviews with patients and their families	4.32 ± 1.00	0.23	0.63	7.3.2 Actively take actions for treatment and rehabilitation	4.74 ± 0.56	0.12	0.79
7.1.2 Behavioral observation (helplessness, self-abandonment, etc.)	4.21 ± 0.98	0.23	0.58	7.4 Intervention plan for helplessness	4.79 ± 0.42	0.09	0.79
7.1.3 Use a scale* for initial screening and determine the degree of helplessness	4.16 ± 0.96	0.23	0.53	7.4.1 For grade Ⅰ helplessness, use narrative nursing.	4.79 ± 0.54	0.11	0.84
7.2 Establish a counseling relationship	4.79 ± 0.54	0.11	0.84	7.4.2 For grade Ⅱ helplessness, use narrative, mindfulness and peer support.	4.79 ± 0.54	0.11	0.84
7.2.1 Respect the patients	4.26 ± 0.93	0.22	0.58	7.4.3 For grade Ⅲ helplessness, use narrative nursing, peer support, mindfulness meditation and NLP self – integration method.	4.26 ± 0.93	0.22	0.58
7.2.2 Show empathy	4.84 ± 0.38	0.08	0.84	7.5 Effect evaluation	4.89 ± 0.32	0.06	0.89
7.2.3 Introduce psychological support resources	4.68 ± 0.75	0.16	0.79	7.5.1 Self – efficacy is improved, and the score < 5 points.	4.79 ± 0.54	0.11	0.84
7.2.4 Introduce the manifestations and impacts of helplessness	4.53 ± 0.96	0.21	0.68	7.5.2 Actively take self – management behaviors.	4.26 ± 0.93	0.22	0.58

Note: *Inpatient Psychological Issues Initial Screening Scale.

## Discussion

### The scientific nature and general applicability of the intervention protocol

This study is based on the empowerment theory and comprehensively searched for relevant content on psychological intervention for elderly patients with chronic diseases. Based on the included literature and the results of the current situation survey, a preliminary intervention plan was formulated. Subsequently, two rounds of Delphi expert consultations were conducted following strict procedures to further refine the plan. The consultation results were systematically summarized to ensure the scientific and feasibility of the intervention plan. The selection criteria for the experts in the consultation were standardized, and 19 experts from the fields of psychological care and nursing management were recruited. All experts had over 10 years of working experience, and 89.5% held senior titles, indicating good representativeness. The participation rate of the experts in both rounds of consultation was 100%, indicating high enthusiasm. The consistency coefficient (Cr) values of the two rounds of consultation were 0.86 and 0.87, both greater than 0.80, indicating that the experts had high authority. In the second round of consultation, the W value was 0.123 (P < 0.001), and all CV values were less than 0.25, indicating that the experts’ opinions were becoming more consistent [13]. Therefore, based on the above favorable objective indicators, the revised plan has stronger theoretical support and provides a solid foundation for subsequent implementation.

### The specificity and effectiveness of the intervention protocol

This program is jointly developed by a multidisciplinary team including clinical nurses and psychotherapists, closely integrating the physical and mental characteristics of elderly patients with chronic diseases who are often accompanied by negative emotions such as anxiety and depression [14].The program integrates empowerment theory with psychological intervention practice, and through a systematic empowerment process, stimulates the patients’ internal potential, effectively relieves their psychological stress, and improves treatment compliance [15,16].

In view of the particularities of elderly patients with chronic diseases, this program has made corresponding adjustments in terms of intervention duration and content design. In terms of time arrangement, considering the weak physical function of elderly patients, the duration of a single course is strictly controlled within 30–40 minutes to avoid fatigue [17]. In terms of intervention content, based on the actual situation of the gradual decline in cognitive function of the elderly population, psychological practical techniques that are easy to understand and master, such as music therapy [18], mindfulness intervention [19], and relaxation training [20], are selected. In addition, the program also adopts differentiated and hierarchical progressive intervention strategies according to different types of psychological problems of patients, so as to more accurately meet individual needs. Therefore, this intervention program has both pertinence and specificity, fully reflecting the “patient-centered” nursing concept, and is helpful to guide medical staff to implement standardized and systematic psychological intervention for elderly patients with chronic diseases.

## Limitations

This study has certain limitations. First, after two rounds of expert consultation, the Kendall’s coefficient increased, but the degree of coordination of expert opinions was still relatively low. Second, elderly patients with Chronic diseases have poor physical condition and Cognition, and may be difficult to fully follow the intervention plan, which may affect the evaluation of intervention effect. In addition, this study only constructed a program framework and did not conduct a pilot test, and its feasibility and Effectiveness need to be verified through subsequent excess syndrome/pattern studies (such as randomized controlled trials). Future research should focus on the implementation and effect evaluation of the program.

## Conclusion

This study is based on the empowerment theory and adopts a combined approach of cross-sectional survey, literature review, and Delphi expert consultation. It has formulated a psychological intervention plan for elderly patients with chronic diseases in general hospitals. This plan provides a standardized process for future psychological care of elderly patients with chronic diseases. In the future, further randomized controlled trials can be conducted to verify the effectiveness of this plan.

## Supporting information

S1 File Minimal data set. (XLSX)
